# Geometric quenching of orbital pair breaking in a single crystalline superconducting nanomesh network

**DOI:** 10.1038/s41467-018-07778-7

**Published:** 2018-12-21

**Authors:** Hyoungdo Nam, Hua Chen, Philip W. Adams, Syu-You Guan, Tien-Ming Chuang, Chia-Seng Chang, Allan H. MacDonald, Chih-Kang Shih

**Affiliations:** 1 Department of Physics, The University of Texas at Austin, Austin, TX 78712 USA; 2 Department of Physics, Colorado State University, Fort Collins, CO 80523 USA; 3 Department of Physics and Astronomy, Louisiana State University, Baton Rouge, LA 70803 USA; 4 Institute of Physics, Academia Sinica, Nankang, 11529 Taipei, Taiwan

## Abstract

In a superconductor Cooper pairs condense into a single state and in so doing support dissipation free charge flow and perfect diamagnetism. In a magnetic field the minimum kinetic energy of the Cooper pairs increases, producing an orbital pair breaking effect. We show that it is possible to significantly quench the orbital pair breaking effect for both parallel and perpendicular magnetic fields in a thin film superconductor with lateral nanostructure on a length scale smaller than the magnetic length. By growing an ultra-thin (2 nm thick) single crystalline Pb nanowire network, we establish nm scale lateral structure without introducing weak links. Our network suppresses orbital pair breaking for both perpendicular and in-plane fields with a negligible reduction in zero-field resistive critical temperatures. Our study opens a frontier in nanoscale superconductivity by providing a strategy for maintaining pairing in strong field environments in all directions with important technological implications.

## Abstract (web summary)

It is highly desired to maintain superconductivity in presence of magnetic fields but it is by far difficult. Here, Nam et al. create a single crystalline superconducting Pb nanowire network, where critical temperatures are maintained and critical fields are enhanced in either parallel or perpendicular fields.

## Introduction

Fueled by the rapid advances in nanoscale materials engineering, nanoscale superconductivity has emerged^1–37^ in recent years as an exciting frontier in which nanostructure length scales can be comparable to the two intrinsic superconductor length scales, the penetration depth and the coherence length. In ultra-thin films the usual orbital pair breaking effect of an in-plane magnetic field can be nearly completely suppressed, allowing the Zeeman pair breaking effect to determine the critical field, i.e. the field threshold at which superconductivity is lost. Recently, it was demonstrated that Zeeman pair breaking is also quenched in systems with strong spin–orbit coupling, so that $H_{{\mathrm{c}}\parallel }$ can greatly exceed even the Clogston limit ($H_{{\mathrm{c}}\parallel }$≫ *H*_P_)^38–41^. Unfortunately, ultra-thin samples do not provide protection against orbital pair breaking under perpendicular field.

Under a perpendicular field, it has been found that in a lateral nanostructure consisting of superconducting nanoislands, vortices do not form until the magnetic length is smaller than the physical dimension of the lateral nanostructure, and only then does local superconductivity start to be suppressed^14,42,43^. When the nanoislands are isolated, however, they do not maintain long-range phase coherence or support collective charge transport. In this report we ask whether one can engineer a superconducting nanostructure with both parallel and perpendicular critical magnetic fields enhanced, and still maintain long-range phase coherence. Successful development of such a material would have important technological implications. Below we provide a definitively positive answer to this important question. The properties we explore are related to those of nanowire networks, which can in principle be used in a similar way to increase the perpendicular critical magnetic field^4,15,26^. However these mostly consist of granular nanowires and as their thickness decreases their connectivity tends to be compromised, limiting critical temperatures.

**Fig. 1:**
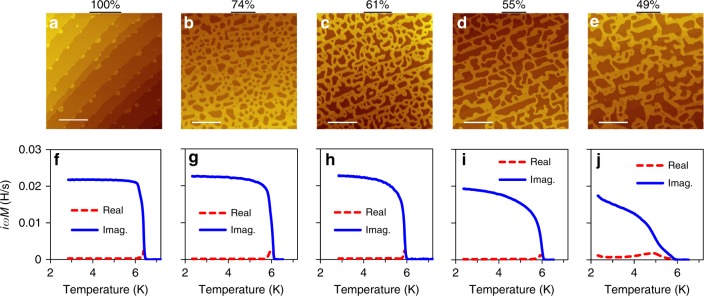
In situ mutual inductance measurements of Pb nanomeshes with different coverages. STM topographic images depending on 7 ML Pb coverage varying from 100% to 49% (**a**–**e**) and in situ mutual inductance measurements on the corresponding samples (**f**–**j**). The real part of the conductivity is a measure of dissipative response, whereas the imaginary part is proportional to the SFD, $n_s \propto \mu _0\omega Y_2/t \equiv 1/\lambda ^2$ (ref. ^46^). Here, *μ*_0_ is the magnetic permeability, $\omega$ an angular frequency, $Y \equiv \left( {\sigma _1 + i\sigma _2} \right)t$ a complex sheet conductivity, *t* a film thickness, and *λ* a penetration depth. The scale bars in (**a**–**e**) represent 500 nm

Here we use an epitaxial approach to create an ultra-thin (2 nm) single crystalline random nanomesh with a distribution of wire widths upward from 10 nm. A series of 2D nanomesh samples were produced by tuning the epitaxial growth kinetics (see Methods). By varying growth conditions, the nanomesh can be tuned to be above or below the percolation threshold. For all nanomesh samples, the desired amount of Pb was deposited on a Pb/Si(111)-striped incommensurate (SIC) wetting layer. (The SIC wetting layer, by itself, has a *T*_c_ of ~1.8 K^44^.) Figure 1a–e is a topographic image of 2D nanomeshes with different coverages (100%, 74%, 61%, 55%, and 49%). The Pb wires are seven monolayers (MLs) thick (~2 nm), measured from the wetting layer. At 74% coverage, the sample can be considered as random distribution of voids in the film. The nanomesh geometry becomes evident in the 61% and 55% samples where nanowires of different widths are well connected. At 49%, which we take as the percolation threshold^45^, connectivity is significantly reduced. In these nanomeshes, one observes elongated voids roughly aligned along the silicon-substrate step edges in the diagonal direction of the topographic images. Atomic images acquired at different locations within the 7 ML region always show the 1 × 1 periodicity of a bulk single crystal with a (111) orientation, whereas the void areas have SIC structure with a mixture of $\sqrt 3 \times \sqrt 3$ and $\sqrt 3 \times \sqrt 7$ periodicities (see Supplementary Figure 1).

## Results

### Superconductor phase rigidity in nanomeshes

To probe the supercurrent response at macroscopic length scale, we performed in situ mutual inductance measurements^46^ on the same sample after STM measurements. Figure 1f–j shows a series of mutual inductance vs temperature curves for coverages ranging from 100% (7 ML Pb film) to 49%. Although nanomeshes with coverages higher than 61% do contain puddles of voids, their superfluid density (SFD) is robust and the temperature range in which dissipation response is observable is similar to the 100% coverage case. At 55% coverage, a small reduction of *σ*_2_ (proportional to the SFD) is accompanied by an enhanced observable dissipative component (*σ*_1_) near *T*_c_. Finally, for 49% nanomesh, in addition to a significant reduction of *σ*_2_, a substantial *σ*_1_ is observed over the entire temperature range below *T*_c_ that can be attributed to the loss of connectivity below the percolation threshold. Another interesting result is that *T*_c_ is around 6 K for all samples suggesting that it is a set by the local Cooper pairing instability rather than by the phase rigidity$V_0 = \frac{{\left( {\hbar c} \right)^2t}}{{16{\mathrm{\pi }}e^2\lambda ^2\left( 0 \right)}}$^47^, where *ħ* is the Plank constant, *c* the speed of light, *e* the elemental charge, *λ*(0) the penetration depth, and *t* a film thickness. Note that due to the limitation of the instrument response, the signal of Im(*iωM*) shown in the lower panel, saturates at ~0.023 H/s. To quantitatively extract the SFD information, the full temperature scan of Im(*iωM*) needs to be below this saturation (e.g. the data for 55% nanomesh). However, as discussed in Supplementary Note 2, the slope of dIm(*iωM*)/d*T* near *T*_c_ is proportional to the SFD. The SFD for the 55% nanomesh sample is determined to be 8/μm^2^ at 2.3 K. Then by comparing the slope of dIm(*iωM*)/d*T* near *T*_c_, we can extract indirectly an SFD of 22/μm^2^ for flat 7 ML film, corresponding to a superfluid rigidity of *V*_0_/*k*_B_ = 500 K, much greater than *T*_c_ ~ 6 K. Similarly, the SFD for the 74% and 61% samples are 18/μm^2^ and 16/μm^2^ respectively. Our nanomeshes derive their strong superconductor phase rigidity from the 2D Pb films, despite the presence of voids.

### Local superconductivity in the nanomeshes

**Fig. 2:**
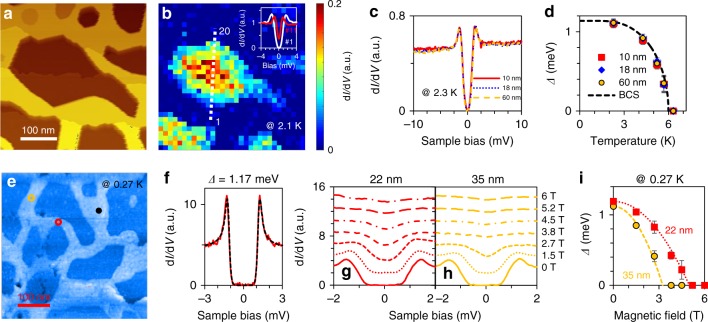
The dependence of local superconductivity on local nanowire widths. **a** STM topographic image with CITS performed at 2.1 K. **b** Zero bias conductance (ZBC) image of **a**, showing spatial variation of superconductivity. The inset is a plot comparing full tunneling spectra at the center of a Pb nanowire and at the center of the void along the white dashed line in **b**. **c**, **d** Tunneling spectra at 2.3 K (**c**) and temperature-dependent superconducting gaps (**d**) for nanowire widths of 10, 18, and 60 nm. **e** STM topographic image of a 3-nm-Ge-capped nanomesh. **f** Tunneling spectrum (red solid) at 0.27 K on the black dot in **e** and the fit (black dash) of the tunneling spectrum with BCS line shape. **g**, **h** Tunneling spectra at 0.27 K as a function of perpendicular magnetic field on 22-nm- and 35-nm-width nanowires marked by red circle and yellow circle in **e**, respectively. **i** Magnetic field dependence of superconducting gaps at 0.27 K, deduced by BCS fitting to the tunneling spectra in **g**, **h** (see Supplementary Table 1 for details). Experimental data were fitted with the equation of $\Delta \left( H \right) \propto 1 - \left( {H/H_{\mathrm{c}}} \right)^2$. Acquired $H_{{\mathrm{c}} \bot }$ are 5 and 3.25 T for 22- and 35-nm-width nanowires, respectively. The error bars in **d**, **i** represent the uncertainty of superconducting gap fitting to tunneling spectra due to noise in the spectra

We use scanning tunneling spectroscopy (STS) to measure the local superconducting gap. Figure 2a is a 400 nm × 400 nm scale topographic image of a 61% sample in which current image tunneling spectroscopy (CITS) was carried out at 2.1 K. Figure 2b is the corresponding zero bias conductance map which captures the local superconductivity variation. Although SIC has *T*_c_ of 1.8 K, at the measurement temperature of 2.1 K the proximity effect can induce superconductivity inside the void. Note that STS data acquired inside this void, shown in inset of Fig. 2b, have noticeably enhanced coherence peaks, which is characteristics of the solution of Usadel equation for the induced gap across the superconductor–normal-metal junction as discussed previously^48,49^. Temperature-dependent STS data were acquired at several representative locations with different local wire widths at 2.3 K, as illustrated in Fig. 2c. Within experimental uncertainty the gaps are identical and their temperature dependences, Δ(*T*) of Fig. 2d, are also identical. A *T*_c_ of ~ 6 K can be extracted from these data, the same as the *T*_c_ value measured using the double coil. The value is also similar to that of a 2D film of the same thickness (~ 6.3 K). This behavior is very different from that for 2D Pb nanoislands on the wetting layer reported previously, which exhibit strong reductions of *T*_c_ when the effective diameter is decreased to below 60 nm^14,22^. This result reflects the fact that these well connected wires have a much stronger superfluid than their 2D island counterparts.

Low-temperature STS measurements under strong perpendicular magnetic fields also show very intriguing results. These spectra are acquired at 0.27 K on a Ge-capped 61% nanomesh sample whose STM image is shown in Fig. 2e. Typical STS data acquired in the 7 ML region with wire width >60 nm is shown in Fig. 2f (marked by the black dot in the image). A Δ value of 1.17 meV is obtained by fitting the spectrum with a BCS line shape, yielding 2Δ/*k*_B_*T*_c_= 4.5, similar to the value for uncapped 2D films and nanomeshes. Magnetic field dependent spectra were acquired in regions with different local wire widths. Shown in Fig. 2g, h are *H*-dependent tunneling spectra acquired in wires of 22 and 35 nm width, respectively. At 0.27 K, the tunneling gaps as a function of *H* at these locations are also plotted, from which perpendicular critical fields of $H_{{\mathrm{c}} \bot }$ = 5.0 and 3.25 T were obtained for the 22 nm wire and the 35 nm wire, respectively (see Supplementary Table 1 for the detailed fitting parameters of the tunneling gaps). Interestingly, the magnetic lengths at the corresponding critical fields ($\ell = \sqrt {\phi _0/\pi H} \sim 26\;{\mathrm {nm}}/\sqrt {H[{\mathrm{T}}]}$, where *ϕ*_0_ is the superconducting magnetic flux quantum) are 11 and 15 nm, respectively, roughly half of the values of the nanowire widths. Previously, Nishio et al.^14^ have reported a similar inverse correlation between $H_{{\mathrm{c}} \bot }$ and the effective diameter of 2D Pb nanoislands (for diameter >60 nm). However, in that case, as the lateral size reduces, *T*_c_ is also reduced rapidly^14,22^. In contrast, here we show that in a nanowire mesh with a random distribution of the wire widths, *T*_c_ is maintained while local $H_{{\mathrm{c}} \bot }$ is inversely correlated with the wire width.

### Extraordinary high ${\boldsymbol{H}}_{{\mathbf{c}} \bot }$ and ${\boldsymbol{H}}_{{\mathbf{c}}\parallel }$

**Fig. 3:**
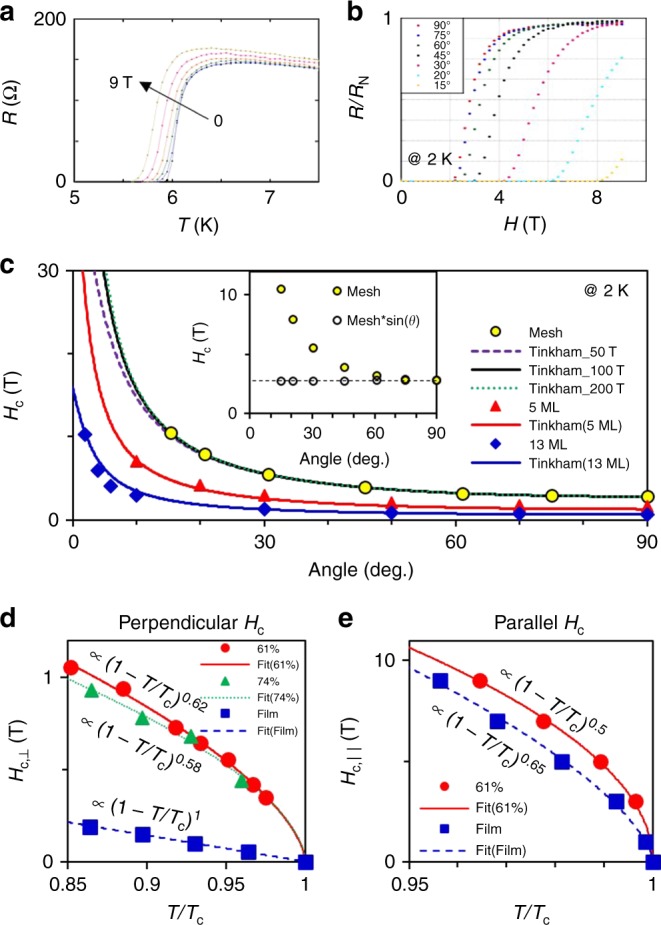
Behaviors of parallel and perpendicular critical fields. On a 61% nanomesh, **a** resistance vs temperature (*R*–*T*) curves at parallel magnetic fields from 0 to 9 T and **b** angular dependence of resistance vs magnetic field (*R*–*H*) at 2 K. **c** The yellow circle symbols are *H*_c_(*θ*) values from **b** defined by *R* = 0.5 × *R*_N_. The black solid line is a Tinkham formula fit to the nanomesh data with $H_{{\mathrm{c}}\parallel }$ = 100 T and $H_{{\mathrm{c}} \bot }$ = 2.8 T; the fits for or $H_{{\mathrm{c}}\parallel }$ = 50 and 200 T are plotted as violet dash and green dot lines, respectively. The data for 5 ML and 13 ML films were reproduced from ref. ^39^. The inset is a plot of *H*_c_(*θ*) and *H*_c_(*θ*) × sin(*θ*) to the nanomesh data. The dashed line in the inset corresponds to $H_{{\mathrm{c}} \bot }$= 2.8 T. **d** Perpendicular and **e** parallel *H*_c_’s near *T*_c_ for 7 ML Pb film and nanomesh samples. Experimental data were fitted to $H_{\mathrm{c}}\left( T \right) \propto \left( {1 - T/T_{\mathrm{c}}} \right)^\gamma$

Magneto-transport measurements were carried out on the Ge-capped 2D nanomesh samples. Figure 3a illustrates resistance vs temperature (*R–T*) spectra of 61% nanomesh at different parallel magnetic fields *H*_||_ from 0 to 9 T. The *T*_c_ of 6 K at zero magnetic field is consistent with the SFD measurement in the bottom middle panel of Fig. 1. Even when *H*_||_ is increased to 9 T the *T*_c_ is still as high as 5.8 K (only a 3% reduction). And there is little broadening of the resistive superconducting-normal phase transition (δ*T* < 0.2 K), which implies strong pinning of magnetic fluxes through the voids in the nanomesh. The normal state resistance *R*_N_ of the 61% nanomesh is around 150 Ω, much lower than the quantum resistance of $h/4e^2\sim 6.5\;{\mathrm{k}}\, {\mathrm{\Omega}}$. This means that the nanomesh is still an excellent conductor, even though *R*_N_ for the nanomesh is three times that of the 7 ML Pb film (50 Ω) due to the presence of the voids.

We also carried out angular dependent *R* vs *H* measurement at a constant temperature of 2 K. Figure 3b illustrates results for the 61% sample. *H*_c_(*θ*) is taken from the transport data at 0.5 × *R*_N_ at each angle and plotted as yellow circles in Fig. 3c. We extrapolate *H*_c_(*θ*) outside of the accessible field range using the Tinkham formula^50^

$$\left| {\frac{{H_{\mathrm{c}}\left( \theta \right){\mathrm {sin}}\theta }}{{H_{{\mathrm{c}} \bot }}}} \right| + \left( {\frac{{H_{\mathrm{c}}\left( \theta \right){\mathrm {cos}}\theta }}{{H_{{\mathrm{c}}\parallel }}}} \right)^2 = 1,$$

which gives the black solid line in the figure with an $H_{{\mathrm{c}}\parallel }$ ~ 100 T, one order of magnitude higher than the Pauli limit $H_{\mathrm P} \approx$11 T. Although the extrapolation is uncertain, the enormous $H_{{\mathrm{c}}\parallel }$ is consistent with the following observations: (1) The perpendicular critical field $H_{{\mathrm{c}} \bot }$ is 2.8 T at 2 K, two times higher than that of a 5 ML film. Similarly, the data at other angles are also about twice the value of the 5 ML film. Previously based on the *H*_c_(*θ*) for a 5 ML flat film, an $H_{{\mathrm{c}}\parallel }$ > 40 T at 2 K was estimated and attributed to quenching of Zeeman pair breaking due to the strong spin–orbit coupling (SOC) in Pb films^39^. Presumably, Zeeman pair breaking is also quenched here due to strong SOC. The fact that the 61% nanomesh samples show a factor of two higher *H*_c_(*θ*) at all angles is consistent with our estimate of $H_{{\mathrm{c}}\parallel }$ ~ 100 T. (2) If we plot *H*_c_(*θ*)sin*θ* as a function of *θ*, all data points collapse to a single value equivalent to $H_{{\mathrm{c}} \bot }$ (inset of Fig. 3c). This is another way of saying that $H_{{\mathrm{c}}\parallel }$ is enormously high and the corresponding pair breaking effect is strongly quenched.

The critical fields of our nanomeshes grow very rapidly below *T*_c_. Figure 3d shows $H_{{\mathrm{c}} \bot }$ as a function of *T*/*T*_c_ for 100% (7 ML film), 74%, and 61% nanomesh samples, respectively. The experimental data were fitted with the equation:

$$H_{{\mathrm{c}} \bot }\left( T \right) \propto \left( {1 - T/T_{\mathrm{c}}} \right)^\gamma .$$

The linear dependence of $H_{{\mathrm{c}} \bot }$ for a flat 7 ML film (*γ* = 1) indicates a normal orbital (Landau) pair breaking effect. In contrast, both nanomesh samples show *γ* ~ 0 6, which is close to the theoretical value of *γ* = 0.5 expected for orbital pair breaking by a parallel critical field. This behavior establishes that at least at high temperature, the orbital pair breaking effect of perpendicular fields on 2D nanomeshes is also quenched, similar to the behavior of $H_{{\mathrm{c}}\parallel }$ shown in Fig. 3e. This is not necessarily of the same origin as the large local $H_{{\mathrm{c}} \bot }$ at low temperatures, however, since the magnetic length in the present case is much larger than the wire widths, so that the magnetic field sees the whole connected network rather than individual wires.

### Linearized Ginzburg–Landau equation with a Gaussian random field

**Fig. 4:**
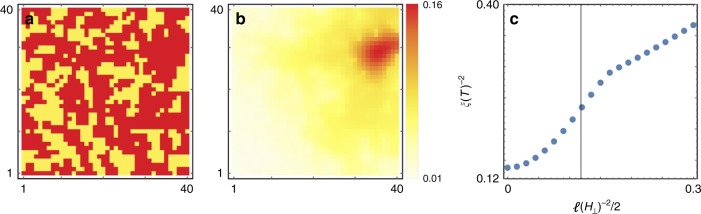
Ginzburg–Landau random *T*_c_ model. **a** Map of a spatially random *T*_c_ on a 40 × 40 square lattice, with the lattice constant set to 1. The dark and light colors correspond to Pb and SIC regions, respectively. The Pb coverage is equal to 61%. The map is generated with a 2D Gaussian random field with correlation length *l* *=* 1. **b** Norm of the wavefunction belonging to the lowest eigenvalue of the linearized Ginzburg–Landau equation on the same lattice at zero magnetic field for $\xi _0$ = 1/3. **c** Dependence of $\xi ^{ - 2}$ $\left( { \propto T_{{\mathrm{c}}0} - T_{\mathrm{c}}} \right)$, on *ℓ*^-2^/2 $\left( { \propto H_ \bot } \right)$. Note 2*ℓ*^2^ is the variance of the zeroth Landau level wavefunction which is Gaussian. The vertical line indicates the point when 2*ℓ*^2^ is equal to $\zeta ^2$, $\zeta$ being the localization length. $\zeta$ is obtained by fitting (**b**) with a 2D Gaussian function and taking the fourth root of the determinant of the covariance matrix

To shed light on the sub-linear temperature dependence of $H_{{\mathrm{c}} \bot }$ close to *T*_c_, we solve the linearized Ginzburg–Landau equations, valid close to the superconducting-normal phase boundary, for a 2D square lattice model with a spatially random *T*_c_ at zero field. The random *T*_c_ map (Fig. 4a) is generated from a Gaussian random field, with the correlation length *l* larger than the zero temperature coherence length $\xi _0$. The variation of *T*_c_ is set to the difference between the *T*_c_’s of Pb and the SIC regions measured experimentally (see Supplementary Notes 5–8 for details). For incomplete Pb coverage, we find that the eigenvector corresponding to the lowest eigenvalue of the linearized Ginzburg–Landau equation of our model at zero magnetic field is generally localized (Fig. 4b), with a localization length $\zeta$ dependent on the correlation length of the random pairing potential, but varying between different realizations of the random *T*_c_ map. Physically, this means that at high temperatures close to *T*_c_, the superconductivity onset is initially localized in small isolated regions with linear scale $\zeta$ in the random nanomesh. Note this statement only applies at the phase boundary and does not contradict with the global coherence established inside the superconducting phase.

In the presence of a perpendicular magnetic field, solving the linearized 2D Ginzburg–Landau equation is formally identical to solving a Landau level problem for an ordinary 2D electron gas. We therefore choose $\ell ^{ - 2}/2 \propto H_ \bot$ to represent the magnetic field strength (see below). In Fig. 4c we plot $\xi ^{ - 2}(H_ \bot )$, where $\xi ^{ - 2}$ is the inverse square temperature-dependent Ginzburg–Landau coherence length, which is proportional to the critical temperature suppression $(T_{{\mathrm{c}}0} - T_{\mathrm{c}})$. $\xi ^{ - 2}(H_ \bot )$ thus depicts the superconducting-normal phase boundary on the $H_ \bot - T$ phase diagram. The super-linear behavior of $\xi ^{ - 2}$ at small $H_ \bot$ implies that $H_{{\mathrm{c}} \bot }(T)$ is sub-linear in $T_{{\mathrm{c}}0} - T$ for *T* close to *T*_c0_, consistent with the experimental results. At higher fields when the zeroth Landau level cyclotron orbit radius $\left( {\sqrt 2 \ell } \right)$ becomes smaller than the localization length $\zeta$ (indicated by the vertical dashed line in Fig. 4c), a linear behavior is recovered. The ordinary orbital pair breaking mechanism is thus quenched at the phase boundary for small fields with $\ell \gg$ $\zeta$, since Landau orbits cannot fit inside the superconducting regions. $H_{{\mathrm{c}} \bot }(T)$ should therefore be quadratic, similar to $H_{{\mathrm{c}} \bot }(T)$ in nanoislands or $H_{{\mathrm{c}}\parallel }(T)$ in thin films. The same argument explains why $H_{{\mathrm{c}} \bot }(T)$ becomes linear at higher fields with *ℓ* $\gtrsim$ $\zeta$. More detailed theoretical descriptions can be found in Supplementary Notes 5–8.

## Discussion

By epitaxially growing a single crystal ultra-thin Pb nanowire network with uniform thickness (2 nm) but random wire widths, we have created a novel superconductor nanostructure in which critical temperatures are maintained and robustness in the presence of external fields is enhanced. The single crystalline nanomesh maintains strong global phase rigidity, thus retaining resistive and gap *T*_c_ values close to those of a bulk crystal. The ultra-thin geometry, plus strong SOC, not only strongly quenches the orbital and Zeeman pair breaking effects of a parallel magnetic field, leading to an extraordinarily high $H_{{\mathrm{c}}\parallel }$ (likely exceeding 100 T), but also suppresses orbital pair breaking in perpendicular fields until the magnetic length becomes smaller than the local wire width so that vortices can form inside the wires. The interplay between the magnetic length and wire widths establishes an inverse correlation of the local $H_{{\mathrm{c}} \bot }$ with the local wire width at low temperature. This inverse correlation points to the possibility of raising $H_{c \bot }$ to an even higher value in a narrower nanomesh. In addition, the random nanomesh quenches the orbital pair breaking of the perpendicular field at high temperatures due to localization of the superconducting order parameter, and leads to a non-linear scaling law of the global $H_{{\mathrm{c}} \bot }$ vs *T*, in stark contrast to the behavior of a flat film. This work demonstrates that superconductivity pair breaking can be significantly suppressed by nanoscale engineering and opens new strategies to optimize superconducting quantum devices.

## Methods

### Sample preparation

For the growth of 7 ML Pb nanomeshes, we first prepared an SIC wetting layer (WL). Using MBE, over 1.3 ML of Pb was deposited on Si(111)-7 × 7 kept at room temperature in 9 ×1 0^−11^ torr, followed by annealing the sample to 400–450 °C for 4 min to induce Pb–Si surface reconstruction. To grow the nanomeshes, a proper amount of Pb was deposited on the WL kept at 80 K. Next we annealed the Pb film at ~ 250 K to induce a Pb thin film to a 7 ML Pb nanomesh, shown in Fig. 1. For ex situ measurements, the nanomesh samples were capped with 3-nm-amorphous Ge. The 3-nm-amorphous Ge capping layer was deposited on the nanomesh samples at 80 K, followed by room temperature annealing at 30° per hour of temperature increasing rate.

### STM/S and mutual inductance measurements

In situ STM/S and mutual inductance measurements^46^ were carried out on the same samples in an ultrahigh vacuum with ~4 × 10^−11^ torr. Electrochemically etched W tips treated with in situ electron-beam cleaning were used for STM/S measurements. Set-point parameters for STS were 30 pA tunneling current at sample bias +17 mV. The ac-driving current of in situ mutual inductance measurements were 15 μA at 50 kHz. For magnetic field dependence of STS, we used a homebuilt 250 mK STM system with the 7 T magnetic field perpendicular to the sample surface. Set-point parameters for ex situ STS were 45 pA tunneling current at sample bias −6 mV.

### Magneto-transport measurements

For magneto-transport measurement^51^, leads were attached to the Ge-capped nanomesh samples by first depositing Cr/Au contact pads via an e-beam evaporator and then soldering a fine Pt wire to the contact pads with Wood’s metal. The samples were aligned to parallel orientation with an in situ mechanical rotator.

## Supplementary information

Supplementary Information

## Data Availability

All relevant data are available from the corresponding author.
