# Supplementary material for: Geometric quenching of orbital pair breaking in a single crystalline superconducting nanomesh network
Source: Nat Commun. 2018 Dec 21;9:5431. doi: 10.1038/s41467-018-07778-7 (PMC6303408; doi:10.1038/s41467-018-07778-7)
Supplement: Supplementary file 1 — Supplementary Information [file 41467_2018_7778_MOESM1_ESM.pdf]

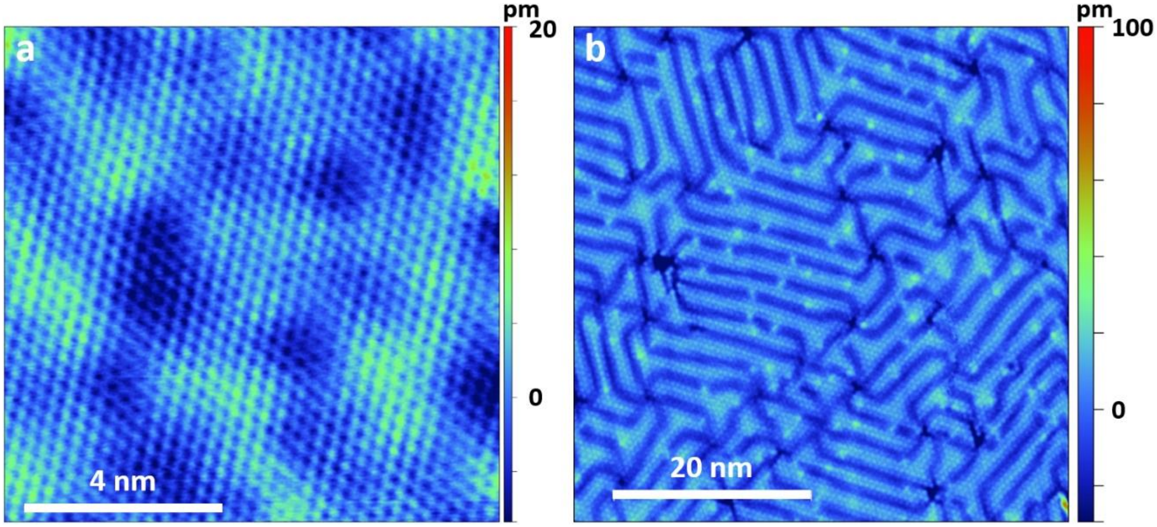

Supplementary Figure 1| Atomic images on Pb wires and in void area. (**a**) in 7 ML region at set-point parameter of (-30 mV, 50 pA) and (**b**) in void area at set-point parameter of (+2 V, 10 pA)

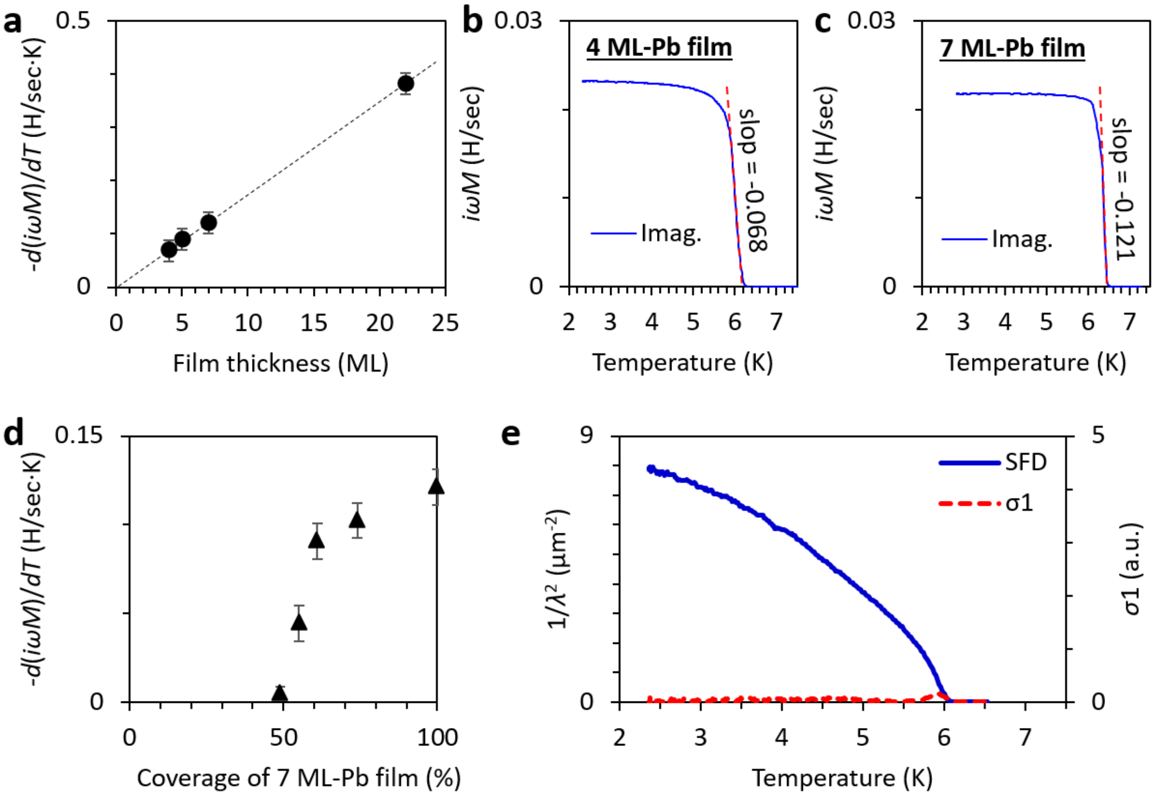

Supplementary Figure 2| Mutual inductance slope near T_c. (**a**) Pb film thickness dependence of the mutual inductance slope. (**b,c**) The way to evaluate the slope of $i\omega M$ for the cases of 4 ML- and 7 ML-Pb films. (**d**) 7 ML-Pb coverage dependence of the mutual

inductance slope. (**e**) SFD of 55 %-manomesh, extracted from the raw data shown in Fig. 1. Error bars in (**a**,**d**) represent the error of linear fit to $i\omega M(T)$ curves near T_c.

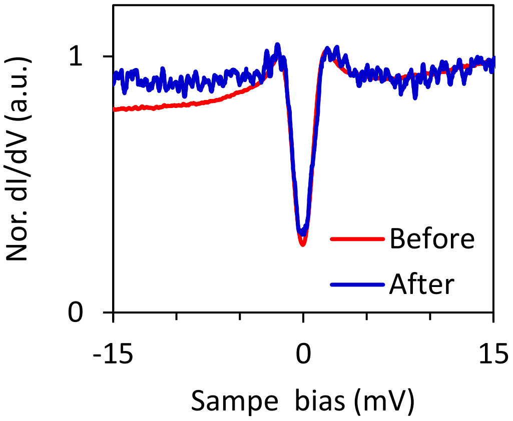

Supplementary Figure 3| Tunneling spectra taken at 4.2 K, before (red) and after (red) Ge-capping.

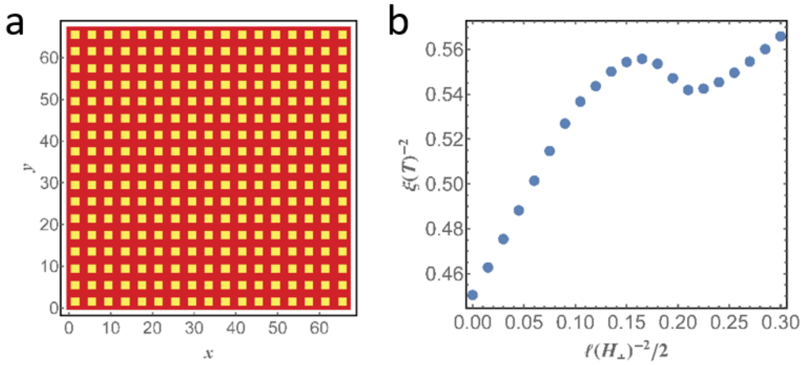

Supplementary Figure 4| A regular network formed by periodic pairing potential. (**a**) Periodic paring potential with its period equal to 4 times the length of the nearest neighbor bond (set to 1) in a regular square lattice. Red and yellow colors correspond to Pb and SIC, respectively. (**b**) Dependence of ξ^{-2} (which is proportional to T_c) on $\frac{\ell^{-2}}{2}$ (which is proportional to $H_\perp$) calculated for the periodically-modulated system in **a** using linearized Ginzburg-Landau theory.

Magnetic field	Width 22nm<br>Fitting results	Width 35nm<br>Fitting results
0 T	$\Gamma = 0.0015$ meV<br>$\Delta = 1.19$ meV	$\Gamma = 0.0018$ meV<br>$\Delta = 1.12$ meV
1.5 T	$\Gamma = 0.07$ meV<br>$\Delta = 1.05$ meV	$\Gamma = 0.16$ meV<br>$\Delta = 0.85$ meV
2.7 T	$\Gamma = 0.15$ meV<br>$\Delta = 0.83$ meV	$\Gamma = 0.25$ meV<br>$\Delta = 0.41$ meV
3.8 T	$\Gamma = 0.25$ meV<br>$\Delta = 0.49$ meV	-
4.5 T	$\Gamma = 0.37$ meV<br>$\Delta = 0.22$ meV	-

Supplementary Table 1| List of superconducting gap fitting parameters as a function of magnetic field.

Supplementary Note 1. Atomic images on Pb wires and in void area

The atomic image shown in Supplementary Fig. 1a for Pb (111) surface always has the same orientation with respect to the Si(111) substrate (albeit with a 10% lattice mismatch). Since the Si substrate being used is single crystalline, single crystallinity of the nanomesh is well maintained.

Supplementary Note 2. The saturation behavior of Im($i\omega M$) and the quantification of superfluid density

To the zeroth order, the imaginary part of the $i\omega M$ corresponds to the imaginary part of sheet conductivity (σ_1+iσ_2) which has been used to determine the superfluid density (SFD). Quantification of SFD requires accurate measurements of σ2(T) over a large temperature range. The saturation behavior of $i\omega M$ due to the limitation of the instrument

response, prevents direct quantification of SFD. Nevertheless, we found that $-d(i\omega M)/dT$ can be used to infer SFD. This is based on the observation that as the SFD increases, the σ2 signal reaches saturation region within a smaller temperature window below T_c. This suggest that the slope of $i\omega M$ can be used to estimate SFD. Indeed, measurement on atomically smooth Pb film, show linear dependence of $-d(i\omega M)/dT$ with respect to the film thickness (shown in Supplementary Fig. 2a). In the case of the measurement on 55% coverage nanomesh sample, the σ2 signal is below the saturation at all temperature. This allows us to quantify the SFD for 55% sample, we then use the slope near T_c to infer SFD for other coverage. The result is shown in Supplementary Fig. 2d. At 2.3K, the SFD for the 55 % nanomesh is ~ 8/μm^2. By using the proportionality of SFD and the $-d(i\omega M)/dT$ near T_c for a flat 7 ML film the SFD is 22 /μm^2 and for 61 % nanomesh, the SFD is ~ 16 /μm^2.

Supplementary Note 3. Scanning tunneling spectroscopy measured at 4.2 K before and after Ge-capping

Since Ge has a small energy gap of 0.7 eV and a low effective mass, the effective tunneling length should be significantly longer than the tunneling decay length in vacuum. Since we have not found the quantitative estimate in the literature, we can estimate the tunneling decay length based on the barrier height (taken at value of 0.35 eV, half of the gap) and an estimated effective mass of 0.3 which will yield a decay constant κ of ~ 0.16 $Å^{-1}$. This is about 1/6 of the decay length in vacuum tunneling. Thus, the tunneling probability across a 3 nm Ge barrier would be similar to the tunneling probability across a 0.5 nm vacuum barrier. Taking a typical vacuum tunneling gap distance of 1 nm, tunneling

through a 3 nm Ge-capped sample would not be a problem in an STM step up. We further experimentally verify this conclusion by carrying out STS of a 5 ML-Pb film before and after in-situ capping in UHV. Shown in Supplementary Fig. 3 is a comparison of the STS measured at 4.2 K before and after Ge-capping. As can be seen, the tunneling spectra are nearly identical.

Supplementary Note 4. Superconducting gap fitting

We fit our superconducting spectrum by the tunneling equation, which includes the Fermi-Dirac distribution function. We also include a phenomenological broadening parameter, Γ which is introduced by Dyne *et al.* [1] so that E → E+*i*Γ. Here, Γ is for the pairing breaking effect due to either the magnetic field or temperature as shown in our fitting and other systems under magnetic field [2, 3]. A rigorous theoretical description on "Dynes formula" in the presence of magnetic field has been discussed in Ref. 4. The values Δ and Γ as a function of magnetic field are listed in the Supplementary Table 1.

Supplementary Note 5. Generation of a 2D Gaussian random field [5]

For a Gaussian random field the joint distribution function is

$$f(X) = \frac{1}{\sqrt{(2\pi)^n detC}} exp\left[-\frac{1}{2}(X-\mu)^T C^{-1}(X-\mu)\right],$$

where $X = (X_1, X_2, \dots, X_n)^T$ represents the values of the random field X at all spatial positions. The vector μ is the mean and the matrix C is the covariance:

$$\mu_i = \langle X_i \rangle,$$

$$C_{ij} = Cov(X_i, X_j) = \langle (X_i - \mu_i)(X_j - \mu_j) \rangle.$$

Note that a Gaussian random field is completely determined by μ and C. Moreover, for a 2D stationary Gaussian field with zero mean μ_i is identically zero, and C_{ij} is only dependent on the distance between x_i and x_j, $\xi = x_j - x_i$. However, it should be noted that $C_{ij} \equiv C(\xi)$ can take various forms (i.e., it is not necessarily a Gaussian function). The common covariance functions are

$$\text{Constant : } C_{ij} = C,$$

$$\text{Linear : } C_{ij} = x_i x_j,$$

$$\text{White noise : } C_{ij} = \sigma^2 \delta_{ij},$$

$$\text{Gaussian : } C_{ij} = \exp\left(-\frac{|\xi|^2}{l^2}\right),$$

Since we need a spatial correlation length we are going to use the Gaussian covariance function. Using this covariance function $C(\xi)$ we can get the spectral density $S(k)$ as

$$S(k) = \frac{1}{(2\pi)^n} \int e^{-ik^T x} e^{-\frac{|x|^2}{2l^2}} = \left(\frac{l}{\sqrt{2\pi}}\right)^n e^{-\frac{|k|^2 l^2}{2}},$$

which is a Gaussian function with variance l^{-2}. Therefore if a random, uncorrelated, zero mean $dZ(k)$ are generated with the variance $S(k)dk$ above, their inverse Fourier transform will give a correlated random Gaussian field $X(x)$ with correlation length l.

In numerical generation of random fields one has to stick with finite random fields on a discrete grid. Moreover, since the spectral representation of a stationary random field is only defined for an infinite domain, it is assumed that the random field is periodic. A $N \times N$ grid in real space will correspond to a $N \times N$ grid in the frequency space. On this grid we have a Fourier series

$$X(x_n) = \sum_m e^{ik_m^T x_n} z(k_m),$$

where m runs through $N \times N$ grid points, and $z(k)$ are random Fourier coefficients with the same properties as $dZ(k)$: 0 mean, variance $\sigma^2 = S(k)\Delta k$, and all $z(k)$ independent for $k_1 \neq k_2$. Furthermore, to ensure $X(x)$ is real, one must have $z(-k) = z^*(k)$, i.e., one only needs $z(k)$ in half of the reciprocal space. In practice $z(k)$ is generated at each grid point m from two independent Gaussian random numbers α_m and β_m, each with 0 mean and variance $\sigma^2 = \frac{1}{2}$ as

$$z(k_m) = \sqrt{S(k_m)\Delta k}(\alpha_m + i\beta_m).$$

A convenient way of generating $N^2/2$ pairs of uncorrelated Gaussian random variables α_m and β_m, as discussed above, is by taking N values at equally spaced grid points of a Gaussian process with zero mean and $\sigma^2 = 1$, and then doing a discrete Fourier transform. The N complex Fourier amplitudes correspond to $2N$ uncorrelated Gaussian variables with zero mean and variance $\frac{1}{2}$.

Supplementary Note 6. Linearized Ginzburg-Landau equation with random pairing potential

The Ginzburg-Landau free energy of a superconductor is

$$F_S = F_N + \int \left[\alpha(T)|\psi|^2 + \frac{\beta(T)}{2}|\psi|^4 + \frac{1}{2m}|(i\hbar\nabla - 2e\mathbf{A})\psi|^2 + \frac{H_i^2}{2}\right] d^3r, \qquad (1)$$

where F_N is the normal state free energy, $\boldsymbol{H}_i$ is the magnetic field induced by the supercurrents and $\mathbf{A}$ is the vector potential with $\nabla \times \mathbf{A} = \boldsymbol{H}_e + \boldsymbol{H}_i$, where $\boldsymbol{H}_e$ is the applied

magnetic field. We choose the Coulomb gauge $\nabla \cdot \mathbf{A} = 0$. The coefficients $\alpha(T)$ and $\beta(T)$ have the form

$$\alpha(T) = C(T - T_\mathrm{c}), \beta(T) = \beta(T_c) = C',$$

where T_c is the zero field critical temperature, C and C' are constants. $\alpha(T)$ and $\beta(T)$ have in addition the following relations

$$\frac{\alpha}{\beta} = -|\psi_\infty|^2, \frac{\alpha^2}{\beta} = H_\mathrm{c}^2,$$

where ψ_∞ is the zero field order parameter and $H_\mathrm{c}(T)$ is the thermodynamic critical field. The Ginzburg-Landau coherence length is defined as

$$\xi = \frac{\hbar}{\sqrt{2m|\alpha|}},$$

Or equivalently,

$$\xi = \frac{\xi(0)}{\sqrt{1-T/T_\mathrm{c}}}.$$

The G-L free energy Supplementary Eq. (1) can now be written as

$$F_{GL} = F_S - F_N = H_c^2 \int \left[-|\psi|^2 + \frac{|\psi|^4}{2} + \xi^2 \left|\left(i\nabla - \frac{2\pi}{\Phi_0}\mathbf{A}\right)\psi\right|^2\right] d^3r + \int \frac{H_i^2}{2} d^3r,$$

where $\Phi_0 = \frac{h}{2e}$ is the magnetic flux quantum, and the rescaled order parameter is ψ/ψ_∞. By letting the variation of F_{GL} vanish we obtain the Ginzburg-Landau equations:

$$\xi^2 \left(i\nabla - \frac{2\pi}{\Phi_0}\mathbf{A}\right)^2 \psi + (|\psi|^2 - 1)\psi = 0, \quad (2)$$

$$\nabla \times (\nabla \times \mathbf{A}) = \mathbf{j},$$

where

$$\mathrm{j} = \frac{\Phi_0}{\lambda^2}\left[\psi^*\left(i\nabla - \frac{2\pi}{\Phi_0}\mathbf{A}\right)\psi + c.c.\right],$$

where $\lambda = \sqrt{m/4e^2|\psi_\infty|^2}$ is the penetration depth. The two equations in Supplementary Eq. (2) are respectively a nonlinear Schrödinger-like equation for the order parameter ψ and a Maxwell equation connecting the magnetic field with the supercurrent density. The coherence length ξ and the penetration depth λ are the characteristic distances for changes in the fields ψ and $\mathbf{A}$ respectively.

At the superconducting-normal phase boundary the nonlinear term in the 1st equation of Supplementary Eq. (2) can be neglected, yielding the linearized Ginzburg-Landau equation

$$\xi^2\left(i\nabla - \frac{2\pi}{\Phi_0}\mathbf{A}\right)^2\psi - \psi = 0,$$

which can be regarded as an eigenvalue problem

$$\left(i\nabla - \frac{2\pi}{\Phi_0}\mathbf{A}\right)^2\psi = \xi^{-2}\psi,$$

where ξ^{-2} is the lowest eigenvalue of the operator $\left(i\nabla - \frac{2\pi}{\Phi_0}\mathbf{A}\right)^2$.

We next discuss how to introduce inhomogeneity in the pairing potential in the linearized G-L equation. Based on the idea of Landau the superconducting phase is stabilized by the negative $\alpha(T)$ when $T < T_\mathrm{c}$. One can thus set T_c to be spatially inhomogeneous, which will make both α and β spatially dependent. The linearized G-L equation is now

$$\left(i\nabla - \frac{2\pi}{\Phi_0}\mathbf{A}\right)^2 \psi = \frac{2mCT_{c0}}{\hbar^2}\left[\eta(\mathbf{r}) - \frac{T}{T_{c0}}\right]\psi,$$

where T_{c0} is a reference critical temperature which can be chosen to be the T_c in the absence of disorder, $\eta \equiv T_c/T_{c0} \in [0,1]$ is a number characterizing the inhomogeneity in the pairing potential.

Using the definition of ξ above equation can be rewritten as

$$\left[\left(i\nabla - \frac{2\pi}{\Phi_0}\mathbf{A}\right)^2 + (1-\eta)\xi(0)^{-2}\right]\psi = \xi(T)^{-2}\psi, \qquad (3)$$

which is still an eigenvalue problem but with a spatially dependent potential term $(1 - \eta)\xi(0)^{-2}\psi$. The critical temperature is suppressed by having regions with smaller T_c, i.e regions in which $\eta < 1$ and by the magnetic field. The critical temperature is reached only when the zero-field uniform system value of $\xi(T)^{-2}$, which increases with decreasing temperature, reaches the lowest eigenvalue of the effective Schrödinger operator. Besides the (superconducting) magnetic length $\ell = \sqrt{\frac{\hbar}{2eH}}$, there are two additional length scales in this problem: One is the zero temperature G-L coherence length of the pristine superconductor, $\xi(0)$ or ξ_0; the other is the characteristic length scale of the (presumably Gaussian) inhomogeneity l. For the G-L theory to be valid one needs to assume $\xi_0 < l$. We also assume the penetration depth to be diverging ($|\psi_\infty| \to 0$) near the critical temperature so that the 2nd equation of Supplementary Eq. (2) can be ignored. We then only need to solve Supplementary Eq. (3) numerically.

Supplementary Note 7. Numerical methods [6]

Assuming a 2D square lattice, we introduce the gauge variables

$$U_x(x,y) = \exp\left(\frac{2\pi i}{\Phi_0}\int_{x_0}^{x} A_x(x',y)dx'\right),$$

$$U_y(x,y) = \exp\left(\frac{2\pi i}{\Phi_0}\int_{y_0}^{y} A_y(x,y')dy'\right),$$

where (x_0, y_0) is an arbitrary point. One can show that

$$\left(i\nabla - \frac{2\pi}{\Phi_0}\mathbf{A}\right)^2 \psi = -U_x^*\partial_x^2(U_x\psi) - U_y^*\partial_y^2(U_y\psi),$$

which has the finite difference form

$$\left(i\nabla - \frac{2\pi}{\Phi_0}\mathbf{A}\right)^2 \psi|_{x_i,y_i} = -\frac{U_{x;i,j}\psi_{i+1,j}+U_{x;i-1,j}^*\psi_{i-1,j}+U_{y;i,j}\psi_{i,j+1}+U_{y;i,j-1}^*\psi_{i,j-1}-4\psi_{i,j}}{a^2} + O(a^2), \quad (4)$$

where

$$U_{x;i,j} = exp\left(\frac{2\pi i}{\Phi_0}\int_{x_i}^{x_{i+1}} A_x(x',y_j)dx'\right),$$

$$U_{y;i,j} = \exp\left(\frac{2\pi i}{\Phi_0}\int_{y_j}^{y_{j+1}} A_y(x_i,y)dy'\right).$$

As mentioned earlier we assume vanishing H_i. For $\boldsymbol{H}_e = H_e\hat{z}$ we can set $\mathbf{A} = H_e x\hat{y}$. With this gauge choice $U_{x;i,j}$ and $U_{y;i,j}$ reduce to

$$U_{x;i,j} = 1,$$

$$U_{y;i,j} = \exp\left(2\pi i\frac{x_i}{a}\cdot\frac{H_e a^2}{\Phi_0}\right).$$

The disorder term $(1-\eta){\xi_0}^{-2}\psi$ is trivially discretized as

$$\left(1-\eta_{i,j}\right){\xi_0}^{-2}\psi_{i,j} = \frac{(1-\eta_{i,j})(\xi_0/a)^{-2}\psi_{i,j}}{a^2}, \quad (5)$$

At the boundary of a finite system the net supercurrent must not have any component perpendicular to the boundary, which indicates

$$\hat{n} \cdot \left(-i\nabla + \frac{2\pi}{\Phi_0}\mathbf{A}\right)\psi|_b = 0,$$

where $\hat{n}$ is a unit vector normal to the surface at the position labeled by subscript b. In terms of the gauge variables introduced above this can be written as

$$[n_x U_x^* \partial_x (U_x \psi) + n_y U_y^* \partial_y (U_y \psi)]_b = 0.$$

We take the left boundary as an example, for which $n_x = -1$ and $n_y = 0$. The boundary condition is discretized as

$$\frac{U_{x;i-1,j}^* \psi_{i-1,j} - \psi_{i,j}}{a} = 0,$$

where i, j label a point on the left boundary. $i - 1$ is a fictitious point on the left side of the left boundary, and it is assumed that $U_{x;i-1,j}^* \psi_{i-1,j} - \psi_{i,j} = \psi_{i,j} - U_{x;i+1,j} \psi_{i+1,j}$. Above equation means that at the boundary of the system one simply needs to remove the terms in Supplementary Eq. (4) that are outside the system region, together with the corresponding number of $\psi_{i,j}$.

We are thus left with a linear eigenvalue problem with N variables, N being the total number of grid points in the system. The linear operator consists of two terms described by Supplementary Eq. (4) (properly modified if at the boundary), and Supplementary Eq. (5). The lowest eigenvalue of this operator corresponds to the inverse square of the finite temperature G-L coherence length at the phase boundary which is

proportional to $(1 - T/T_{c0})$. By plotting this eigenvalue vs. H_e one obtains the $T - H$ phase boundary in the presence of inhomogeneous T_c or pairing potential.

To make direct comparison with experimental results, we choose a critical value η_c of the disorder potential, so that the total area of the regions with $\eta > \eta_c$ is equal to the desired coverage of Pb. Moreover, we note that according to experiments the SIC phase has a $T_c \approx 1.85$ K, which is about 31% of the T_c of a homogeneous Pb film. Therefore we replace the disorder potential η by $\tilde{\eta}$, defined by

$$\tilde{\eta}(\boldsymbol{r}) = \begin{cases} 0.31, & \eta(\boldsymbol{r}) < \eta_c \\ 1, & \eta(\boldsymbol{r}) \geq \eta_c \end{cases}$$

This approximation is valid as long as the thickness of the interface between the Pb and the SIC regions is much smaller than the disorder correlation length *l* and the coherence length ξ_0, which is the case in the experiments.

Supplementary Note 8. For a regular network formed by periodically modulated pairing potential

We emphasize here again that the mechanisms for quenching the orbital pair-breaking effect of a perpendicular magnetic field are different at different length scales. When the magnetic length is much larger than the size of the voids, the magnetic field still sees the whole mesh as a percolated 2D system, and the orbital-pair breaking is through formation of circulating supercurrents in the mesh network. For a regular network formed by periodically modulated pairing potential, the long-wavelength behavior of the linearized Ginzburg-Landau equation is still similar to that of an ordinary 2D electron gas. Therefore the pair-breaking parameter for a perpendicular field is linear in field which leads to the

linear dependence of T_c on $H_\perp$. This is shown explicitly in Supplementary Figure 4. At small fields when the magnetic length is much larger than the modulation period, one still has the expected linear behavior. The nontrivial dependence of T_c on $H_\perp$ only appears when the magnetic length is comparable to the period. Thus at the superconducting-normal phase boundary in the *H*-*T* phase diagram, quenching of the orbital pair-breaking effect at high temperatures must be due to deviation of the long-wavelength behavior of the network from that of ordinary 2DEG. This is the reason why the randomness of the mesh network is critical in this regime. As one moves along the phase boundary to lower temperatures, the magnetic length gradually becomes shorter than the voids, but still larger than the wire width, so that closed loops of supercurrents cannot form by going through several links surrounding a void. This is when the narrow width of the wires quenches the orbital pair-breaking effect, similar to that of a parallel magnetic field in a thin film superconductor.

Supplementary References:

1. Dynes, R.C., Narayanamurti, V. & Garno, J. P. Direct Measurement of Quasiparticle-Lifetime Broadening in a Strong-Coupled Superconductor. *Phys. Rev. Lett.* **41**, 1509, doi: 10.1103/PhysRevLett.41.1509 (1978).
2. Eltschka, M., Jäck, B., Assig, M., Kondrashov, O. V., Skvortsov, M. A., Etzkorn, M., Ast, C. R. & Kern, K. Superconducting scanning tunneling microscopy tips in a magnetic field: Geometry-controlled order of the phase transition. *Appl. Phys. Lett.* **107**, 122601, doi: 10.1063/1.4931359 (2015).
3. Sun, Z., Enayat, M., Maldonado, A., Lithgow, C., Yelland, E., Peets, D. C., Yaresko, A., Schnyder, A. P. & Wahl, P. Dirac surface states and nature of superconductivity in Noncentrosymmetric BiPd. *Nature Comm.* **6**, 6633, doi: 10.1038/ncomms7633 (2015).
4. Herman, F. & Hlubina, R. Microscopic interpretation of the Dynes formula for the tunneling density of states. *Phys. Rev. B* **94**, 144508, doi: 10.1103/PhysRevB.94.144508 (2016).
5. Harter, T. Unconditional and conditional simulation of flow and transport in heterogeneous variably saturated porous media, Ph.D. dissertation, Univ. of Ariz., Tucson (1994).
6. Connectivity and Superconductivity, Edited by Berger, J. & Rubinstein, J., Springer-Verlag, Berlin, doi: 10.1007/3-540-44532-3 (2000).
